# Upregulated expression of *FFAR2* and *SOC3* genes is associated with gout

**DOI:** 10.1093/rheumatology/keac360

**Published:** 2022-06-22

**Authors:** Oliver C Orji, Maria B López-Domínguez, Gabriela Sandoval-Plata, Tamar Guetta-Baranes, Ana M Valdes, Michael Doherty, Kevin Morgan, Abhishek Abhishek

**Affiliations:** Human Genetics, School of Life Sciences, Faculty of Medicine and Health Sciences, University of Nottingham, Nottingham, UK; Department of Medical Laboratory Sciences, College of Medicine, University of Nigeria, Enugu Campus, Enugu, Nigeria; Human Genetics, School of Life Sciences, Faculty of Medicine and Health Sciences, University of Nottingham, Nottingham, UK; Human Genetics, School of Life Sciences, Faculty of Medicine and Health Sciences, University of Nottingham, Nottingham, UK; Academic Rheumatology, School of Medicine, Nottingham City Hospital, University of Nottingham; Human Genetics, School of Life Sciences, Faculty of Medicine and Health Sciences, University of Nottingham, Nottingham, UK; Academic Rheumatology, School of Medicine, Nottingham City Hospital, University of Nottingham; Nottingham NIHR BRC, Nottingham, UK; Academic Rheumatology, School of Medicine, Nottingham City Hospital, University of Nottingham; Human Genetics, School of Life Sciences, Faculty of Medicine and Health Sciences, University of Nottingham, Nottingham, UK; Academic Rheumatology, School of Medicine, Nottingham City Hospital, University of Nottingham; Nottingham NIHR BRC, Nottingham, UK

**Keywords:** gout, *FFAR2*, *SOCS3*, gene expression

## Abstract

**Objective:**

To examine the expression of *Free fatty acid receptor 2* (*FFAR2*) and *Suppressor of cytokine signalling 3* (*SOCS3*) genes in asymptomatic hyperuricaemia (AH), AH with MSU crystal deposition, inter-critical gout and gout flare.

**Methods:**

Study participants (*n* = 120) comprised 34 people with serum urate (SU) <360 μmol/l, 69 with AH ± MSU crystal deposition and 17 with a gout flare. Sixteen of the 17 patients with a gout flare attended a second visit 6–12 weeks later. Gene expression levels were assessed using RT-qPCR and results computed as fold changes (FC) after normalization to the reference gene.

**Results:**

*FFAR2* was significantly upregulated during gout flares (FC = 2.9) compared with normal SU, AH, and AH + MSU crystal deposition (FC = 1.1, *P* < 0.0001 for each comparison). *FFAR2* was also significantly upregulated during inter-critical gout (FC = 1.8) compared with normal SU, AH and AH + MSU (FC = 1.1, *P* < 0.001 for each comparison). *SOCS3* was significantly upregulated during gout flares (FC = 3.4) compared with normal SU, AH, and AH + MSU crystal deposition (FC = 1.1, 1.1 and 1.2, respectively, *P* < 0.0001 for each comparison). *SOCS3* was also upregulated during inter-critical gout (FC = 2.1) compared with normal SU (*P* = 0.02) and AH (*P* = 0.006) (FC = 1.1 and 1.2, respectively). *FFAR2* expression was upregulated during gout flare compared with inter-critical gout and *SOCS3* expression showed negative correlation with flare duration (r = –0.49, *P* < 0.05).

**Conclusion:**

*FFAR2* upregulation is associated with gout and may trigger gout flares. *SOCS3* may have a role in amelioration of gout flares.

## Graphical Abstract

**Figure:**
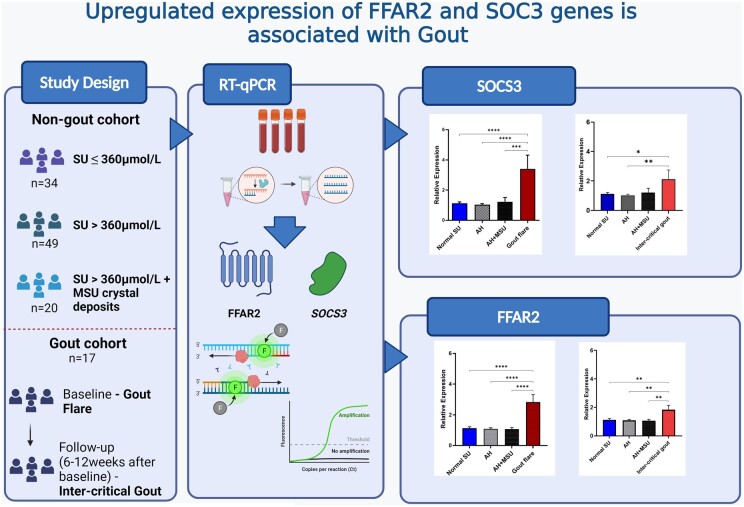

Rheumatology key messages

- *FFAR2* may have a role in the onset of gout flares in those with pre-existing MSU crystal deposits.
- Upregulated *SOCS3* expression during early stages of gout flare, likely part of a negative feedback loop, indicates a potential role in flare resolution.

## Introduction

The clinical phenotype of gout is variable with intermittent unpredictable flares characterized by pain, swelling, erythema and tenderness which result from an inflammatory response to MSU crystals. Even though hyperuricemia is the major risk factor for gout [1], it does not fully explain its pathogenesis and phenotypic variability. A greater understanding of the mechanisms that contribute to the onset of gout flares is required to develop targeted treatments to prevent gout flares.

MSU crystals cause inflammation by activating the NLRP3 inflammasome, which cleaves pro-IL1β to form IL-1β [2]. Recent studies have characterized several regulators of inflammation. *Free fatty acid receptor 2* (*FFAR2*) was identified as a receptor on neutrophils that interacts with short-chain fatty acids (SCFA) such as acetate and propionate and is involved in the regulation of inflammatory gene expression [3, 4]. The Janus kinase/signal transducers and activators of transcription (JAK/STAT) pathway also plays an important role in inflammation [5], and its inhibition reduced disease activity in autoimmune inflammatory arthritis [6, 7]. S*uppressor of cytokine signalling 3* (*SOCS3*) is of interest as it is an important inhibitor of the JAK/STAT pathway [8].

Therefore, the objectives of this study were to examine the expression of *FFAR2* and *SOCS3* genes in people with normal serum urate (SU), asymptomatic hyperuricemia (AH), AH with asymptomatic MSU crystal deposition (AH + MSU), inter-critical gout and gout flare. Additionally, we also explored their association with the duration of gout flare.

## Methods

### Study design

Cross-sectional and prospective cohort study designs were used.

### Participants and recruitment

One hundred and twenty participants were included in this study. They had been recruited as part of the Sons of Gout study, and another prospective study of biomarkers of gout flare. Details of the Sons of Gout study have been published elsewhere [9]. In brief, the study recruited asymptomatic sons of people with gout who attended a single study visit at which targeted musculoskeletal assessment and ultrasonography were performed, and blood and urine samples were collected. Participants that gave peripheral blood for RNA extraction (*n* = 103) were included in the present study. This study was approved by Nottingham NHS Research Ethics Committee (Ref: 15/EM/0316) and all participants gave written informed consent.

Seventeen people currently experiencing gout flare, either crystal proven or with tophaceous deposits, were recruited from inpatient wards of the Nottingham University Hospitals NHS Trust. Information about disease and demographic characteristics and peripheral blood for RNA extraction were collected. Participants attended a research visit 6–12 weeks later and gave another blood sample for RNA extraction. Participants with autoimmune rheumatic diseases, those prescribed immune suppressive treatment, or treated with CS for ≥3 days prior to their baseline visit were ineligible for this study. This study was approved by the Hampshire A NHS Research Ethics Committee (Ref: 15/SC/0730) and all participants gave written informed consent.

Participants included in the current study were classified as:

- Normal SU: SU ≤360 µmol/l (*n* = 34)
- AH: SU >360 µmol/l and no MSU crystal deposition on ultrasonographic examination of target joints (*n* = 49)
- AH + MSU crystal deposition: SU >360 µmol/L and with ultrasound evidence of MSU crystal deposition (*n* = 20)
- Gout flare (*n* = 17): Sixteen participants subsequently gave samples during the inter-critical period and comprised the inter-critical gout group.

### RNA extraction, cDNA synthesis and real-time PCR

Total RNA was extracted using PAXgene Blood RNA Kit (Qiagen, Hombrechtikon, Switzerland) according to the manufacturer’s guidelines. Reverse transcription was carried out with 0.5 µg of RNA using RT^2^ First Strand Kit (Qiagen, Manchester, UK), as recommended by the manufacturer. Real-time PCR was performed with SYBR Green (Qiagen, Manchester, UK) and specific primers for *Ribosomal protein lateral stalk subunit P0* (*RPLP0*) (reference gene), and *FFAR2* and *SOCS3* (target genes) (Qiagen, Manchester, UK). Reactions were performed in triplicate. Gene expression was determined using the comparative C_T_ (ΔΔC_T_) method [10].

### Statistical analyses

Between-group comparisons were carried out using one-way ANOVA with Bonferroni *posthoc* test for pairwise comparison and multiple corrections. Two separate one-way ANOVA analyses were performed—one that included data from normal SU, elevated SU ± MSU crystal deposition and gout flare, and another that included data from normal SU, elevated SU ± MSU crystal deposition and inter-critical gout. Paired *t*-test was used to analyse differences in gene expression between acute and inter-critical gout. Results were reported as mean fold changes (FC) (s.e.m.). Correlations between gene expression and gout flare duration were explored using Pearson or Spearman correlation coefficient as appropriate after testing the data for normal distribution using Shapiro–Wilk test. *P* < 0.05 after Bonferroni correction was defined as statistically significant. All analyses were performed using SPSS version 25 and Graphpad Prism 7.

## Results

The mean (s.e.m.) age was 48 (2.0), 45 (10.3), 47 (9.5) and 68 (13.9) years for participants with normal SU, AH, AH + MSU crystal deposition and gout, respectively. Gout patients were older and had higher BMI than other groups (Table 1). The AH ± MSU crystal deposition group had higher SU than gout patients.

**Table 1:** Demographic characteristics (*N* = 120)

	Normal SU (*n* = 34)	AH (*n* = 49)	AH + MSU crystal deposits (*n* = 20)	Gout (*n* = 17)
Age (years)	48 (2.0)	45 (2.0)^##^	47 (2.0)	68 (4.0)^#^
BMI (kg/m²)	26.31 (1.0)*	27.37 (0.6)	26.88 (0.7)*	29.48 (4.6)
SU (µmol/l)	310.20 (6.76)	429.92 (6.40)**	423.25 (11.9)**	397.71 (28.0)**

Data were obtained from subjects with normal SU levels (*n* = 34), patients with AH (*n* = 49), patients with AH with MSU crystal deposition (AH + MSU) (*n* = 20), and gout flare patients (*n* = 17). Values are expressed as mean (s.e.m.). Differences in means expressed as *P*-values were determined using one-way ANOVA. Variations within the groups were statistically significant for age (*P* < 0.001), BMI (*P* = 0.02) and SU (*P* < 0.001). Tukey’s *post hoc* test was used for pairwise comparison (*P < 0.05 was significant *vs* gout group; **P < 0.05 *vs* normal SU controls; ^#^*P* < 0.05 *vs* normal SU and AH; ^##^*P* < 0.05 *vs* AH+MSU crystal deposits). AH: asymptomatic hyperuricaemia; SU: serum urate.

### *FFAR2* and *SOCS3* are upregulated during gout flare and inter-critical gout

**Fig. 1:**
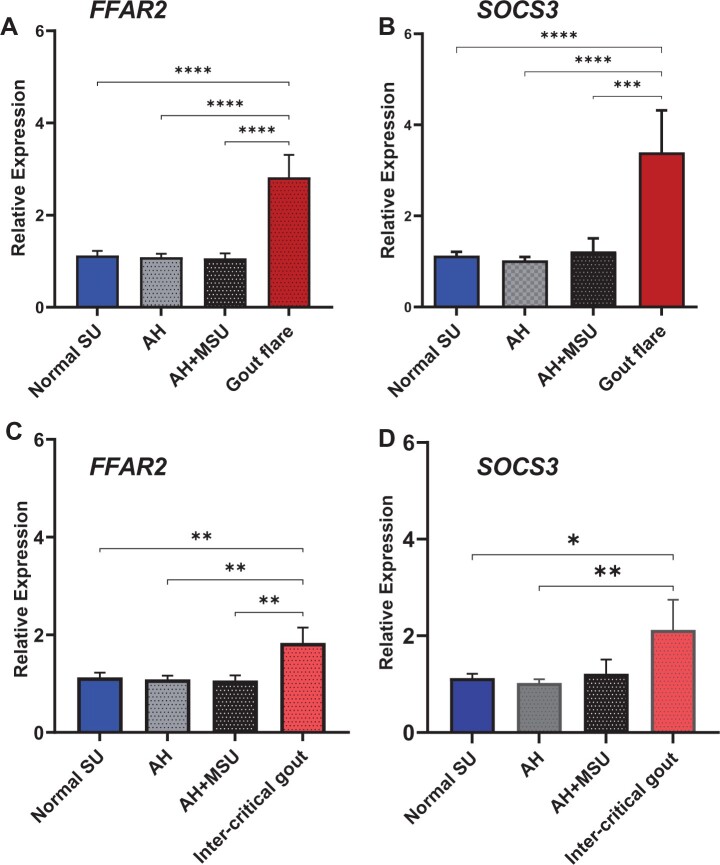
Expression of *FFAR2* and *SOCS3* is increased in patients with gout flare and during the inter-critical period Gene expression was carried out using RT-qPCR normalized to *RPLP0*. Relative expressions are presented as mean fold changes (s.e.m.). (**A**) *FFAR2* expression was increased by 2.9-fold in gout flare patients (*n* = 17) compared with the non-gout groups—normal serum urate (*n* = 34), AH (*n* = 49) and AH+MSU (*n* = 20) (*P* < 0.0001). (**B**) Similarly, *SOCS3* expression was increased by 3.4-fold in gout flare when compared with normal serum urate, AH and AH + MSU (*P* < 0.0001 for each). (**C**) *FFAR2* remained upregulated in inter-critical gout (*n* = 16) by 1.8-fold compared with normal serum urate (fold change = 1.1, *P* = 0.004), AH (fold change = 1.1, *P* = 0.001) and AH + MSU (fold change = 1.1, *P* = 0.005). (**D**) *SOCS3* expression remained upregulated in inter-critical gout by 2.1-fold compared with normal SU (fold change = 1.1, *P* = 0.02) and AH (fold change = 1.0, *P* = 0.01). Fold changes between non-gout groups and gout flare and inter-critical gout groups, respectively, were compared using one-way ANOVA with Bonferroni correction for multiple testing. ****P < 0.0001 was significant with respect to the gout flare group. **P < 0.004 and *P = 0.02 with respect to the inter-critical gout group. *FFAR2*: *Free fatty acid receptor 2*; *SOCS3*: *Suppressor of cytokine signalling 3*; AH: asymptomatic hyperuricaemia; SU: serum urate.

The expression of both genes remained constant at low level in peripheral blood mononuclear cells (PBMC) of individuals with normal SU, AH and AH + MSU crystal deposits [FC (s.e.m.) (*FFAR2*) *=* 1.1 (0.1), 1.1 (0.1), 1.1 (0.1) and (*SOCS3*) = 1.1 (0.01), 1.0 (0.01), 1.2 (0.02), respectively]. *FFAR2* was upregulated [FC (s.e.m.) = 2.9 (0.5)] during gout flares when compared with normal SU, AH and AH + MSU crystal deposition (*P* < 0.0001) (Fig. 1A). The expression of this gene in inter-critical gout patients remained significantly raised [FC (s.e.m.) = 1.8  (0.3)] compared with normal SU (*P* = 0.004), AH (*P* = 0.001) and AH + MSU (*P* = 0.005) (Fig. 1C), but reduced significantly compared with expression during gout flare (*P* = 0.02) (see supplementary Fig.S1, available at *Rheumatology* online). Similarly, *SOCS3* mRNA was 3-fold upregulated [FC (s.e.m.) = 3.4 (0.5)] in patients with gout flares compared with normal SU, AH and AH + MSU (*P* < 0.0001) (Fig. 1B) and remained significantly upregulated during inter-critical gout [FC (s.e.m.) = 2.1  (0.6)] when compared with normal SU (*P* = 0.02) and AH (*P* = 0.006) (Fig. 1D). No significant difference was found in gene expression when gout flare and inter-critical gout were compared (*P* = 0.196) (supplementary Fig.S1, available at *Rheumatology* online).

### *SOCS3* and *FFAR2* expression during gout flare and inter-critical gout

*FFAR2* gene expression showed no correlation with number of flares since onset of gout flares (supplementary Fig.S2A, available at *Rheumatology* online). Conversely, the expression of *SOCS3* showed a statistically significant negative correlation with number of flares since flare onset (supplementary Fig.S2B, available at *Rheumatology* online).

## Discussion

This study reported on the expression of *FFAR2* and *SOCS3* genes in PBMCs during different pre-clinical, and clinical states in the gout hyperuricemia spectrum. It showed that these genes are significantly upregulated during gout flares and in inter-critical gout compared with the pre-clinical stages of gout. Additionally, *FFAR2* expression was significantly increased during gout flares compared with inter-critical gout, and *SOCS3* gene expression was significantly increased early during gout flares.

*FFAR2* expression was increased during inter-critical gout compared with normal SU, AH and AH + MSU crystal deposition. The expression increased further during gout flares compared with inter-critical gout. These findings raise the possibility that *FFAR*2 may have a role in the onset of gout flares. MSU crystal induced activation of the inflammasome requires activation of toll-like receptors by co-stimulatory stimuli such as free fatty acid (FFA) [11]. Additionally, SCFAs induce neutrophil recruitment via the activation of *FFAR2* [12]. This ligand–receptor interaction may represent a key signal for the development of inflammatory response in gout as *FFAR2*-deficient mice (GPR43^–/–^) showed reduced neutrophil recruitment and poor assembly of the inflammasome upon injection with MSU crystals compared with the wild type [4]. A deficiency of *FFAR2* on macrophages led to a reduced activity of caspase 1, which cleaves IL-1β in response to the activation of the NLRP3 complex [4]. Although the interaction between SCFAs and host cells has been analysed mainly in the intestinal lumen [13], recent studies indicate that SCFAs also modulate the function of innate immune cells such as neutrophils, monocytes or macrophages in other tissues and in the blood [14]. A role for acetate (one of the SCFA ligands of *FFAR2*) in triggering inflammatory responses via NLRP3 has been demonstrated recently [15]. Therefore, an involvement of *FFAR2* activation necessary for the onset of the inflammatory response of gout could be explained by the same mechanism [4]. Evidently, SCFAs, including acetate appear to have a complex role both in onset and resolution of MSU crystal-induced inflammation [4, 16–18].

Endogenous signals like *SOCS3* have a critical role in modulating acute inflammation by inhibiting the JAK/STAT pathway [5]. Indeed, our results showed upregulation of *SOCS3* early during gout flares. *SOCS3* is an inducible endogenous regulator of cytokine response through the inhibition of JAK/STAT signalling via a negative biofeedback loop, and this may explain increased gene expression early during a gout flare [8]. This is consistent with the finding that *SOCS3* induction in mouse macrophages stimulated with MSU crystals was associated with resolution of inflammatory events [19]. We found an inverse correlation between *SOCS3* expression and duration of flares, supporting a negative feedback role for the gene in the resolution of inflammation through the inhibition of pro-inflammatory cytokines. *SOCS3* may also mediate anti-inflammatory effects by stimulating the production of anti-inflammatory cytokines such as TGF-β1 [19]. Conversely, other anti-inflammatory cytokines such as IL-37 may also exert their immunosuppressive effects and hence flare resolution by activating *SOCS3* [20]. There was no significant reduction in *SOCS3* expression in inter-critical gout compared with gout flare, which may be due to the small sample size. Additional larger studies are recommended to investigate this further.

This study is limited by several factors. First, we had a relatively small sample size which limited the power to detect smaller differences in gene expression. Another limitation is that we did not perform a parallel analysis of gene expression in the SF.

In conclusion, we have reported on the expression of *FFAR2* and *SOCS3* in the PBMCs of people with pre-clinical and symptomatic phases in the gout hyperuricemia spectrum. *FFAR2* gene expression was increased in inter-critical gout and increased further during a gout flare. This indicated a role in triggering gout flares. *SOCS3* upregulation supports the potential role of this gene in flare resolution. These findings provide an interesting molecular clue to gout pathogenesis and support further research in this area. They also infer that dietary and lifestyle changes may have an important role in preventing gout flares.

## Supplementary Material

keac360_Supplementary_Data

## Data Availability

The data underlying this research will be shared on reasonable request to the corresponding author.
