# Supplementary material for: Upregulated expression of FFAR2 and SOC3 genes is associated with gout
Source: Rheumatology (Oxford). 2022 Jun 22;62(2):977–83. doi: 10.1093/rheumatology/keac360 (PMC9891400; doi:10.1093/rheumatology/keac360)
Supplement: keac360_Supplementary_Data [file keac360_supplementary_data.docx]

**Supplementary** **Figure S1. Differential expression of *Free fatty acid receptor 2 (FFAR2)* and *Suppressor of cytokine signalling (SOCS3)* in gout flares and inter-critical gout**. Gene expression in PMNCs was carried out using RT-qPCR normalized to the reference gene *RPLP0*. Relative expressions are presented as mean fold changes ± SEM. **(A)** *FFAR2* was upregulated 2.9 folds during gout flare (n = 17) and 1.8-fold during inter-critical gout (n = 16). **(B)** Expression of *SOCS3* was similar in acute and inter-critical gout (p = 0.196). Mean fold changes between gout flare and inter-critical gout were compared using paired t-test. **p = 0.02; ns = non-significant; RPLP0 – Ribosomal protein lateral stalk subunit P0; PMNCs – Peripheral blood nuclear cells.

**Supplementary Figure S2.** **Relationship of FFAR2 and SOCS3 expression and duration of gout flares (n = 17).** Relative gene expression, reported as fold changes, were determined using RT-qPCR and normalized to the *Ribosomal protein lateral stalk subunit P0 (RPLP0)*. Duration of gout flares were self-reported as the number of days between the onset of gout flares and sample collection. There was no statistically significant correlation between *FFAR2* expression and duration of flares, while *SOCS3* expression was inversely correlated with the duration of flares. Correlation analysis was performed with Pearson coefficient (r) as data were normally distributed, see methods; r and p-values are shown on the graphs.
